# Needed Medical Legislation

**Published:** 1885-07

**Authors:** 


### NEEDED MEDICAL LEGISLATION.

The Medical Association of the State of Georgia raised a committee to call the attention of the General Assembly to and invoke its action on two subjects of interest to the medical profession.

Bills in relation to them have been introduced, referred to appropriate committees, favorably reported on, and are now pending before that body. The object of one of them is to provide for the payment of reasonable compensation to witnesses sworn as experts in judicial trials; the other, to facilitate the study of anatomy, by permitting, under proper instructions, the procurement of the necessary material for that purpose.

Physicians and surgeons are often put to great inconvenience and loss of time and money by compulsory attendance upon protracted trials to give opinions as experts in cases in which they know nothing of the facts involved. Sometimes in criminal cases they are compelled to attend courts in counties remote from their residences at great personal inconvenience, for which the law provides no adequate compensation. The injustice of this demand for gratuitous professional service is generally admitted, and the law now before the Legislature proposes to remedy it by adopting the usage of several of the States, providing that, in cases where such expert testimony is demanded, the Judge before whom the cause is tried shall determine the sum proper to be paid to the expert witness, to be collected as other court costs.

The other law in relation to anatomical material is even more important. Everybody knows that it is impossible to educate surgeons and physicians without an adequate supply of such material. The statutes of the State passed in the interest of the public health require a thorough and minute knowledge of anatomy of all who practice the healing art, and visit with heavy penalties any who seek to evade the requirement, but most absurdly they punish with great severity any attempt to acquire that knowledge by the only practicable method.

Human anatomy can only be learned by the dissection of human bodies. If these cannot be obtained in Georgia, all proper teaching of medical science must be driven from the State.

There exists in the public mind a very strong aversion to the mutilation of the bodies of friends or acquaintances after death, and to the desecration of graves or cemeteries. With this sentiment it is useles to argue, and it is not proper to outrage it.

Other enlightened communities have found a solution of the difficulty by enacting laws similar to the one now before the Legislature. Scientific pursuits are sufficiently encouraged and their good results secured, and at the same time the sanctity of the proper habitation of the dead securely guarded.

Another subject which deserves the attention of the General Assembly, and demands prompt and well-considered action, is the hygienic condition of the jails and prisons of the State.

### ITEMS.

DR. C. F. BENSON, JR., Demonstrator of Anatomy in the Atlanta Medical College, has resigned, and Dr. F. W. McRae, of Talbotton, has been elected to fill the vacancy.

A COMPLIMENT.—Dr. B. B. Brown, of Spring Place, Ga., says: "I derive a great deal of valuable information from THE JOURNAL under its present able editorial management."

DR. R. J. NUNN, of Savannah, President-elect of the Medical Association of Georgia, has an interesting letter in this issue on locating the Association. The committee appointed to consider this matter should give this letter careful consideration.

A NEW JOURNAL.—Dr. F. E. Daniel, of Austin, Texas, has sold his interest in the *Courier-Record*, of Fort Worth, and will start a new journal in Austin, in July, to be known as *Daniel's Medical Journal*. We wish him success in his new enterprise.

DR. C. D. SMITH, a prominent physician of Newnan, Ga., and a member of the State Medical Association, was in the city a few days ago. He expressed himself as highly pleased with the action of the Association in publishing the Transactions in THE JOURNAL.

THE cholera continues to spread in Spain with much rapidity. The latest reports regarding Ferran's inocculations show them to be an utter failure. The *Medical Record* suggests that Dr. Ferran is only inocculating his countrymen with a mild type of septicæmia.

THE latest advices from German laboratories report no new bacilli this week. There has also been no re-christening of the cholera germ. It is now in order for some one to show that Koch, and all the members of both commissions (French and English), are in entire harmony.—*Canada Medical Record.*

GOVERNOR HILL, of New York, vetoed the bill recently passed by the Legislature appropriating $15,000 to the State Board of Health. Short-sighted policy, we think.

THE Annual Report of the Thomas Wilson Sanitarium for Children, of Baltimore city, under charge of Dr. Wm. D. Booker, shows that excellent charity has been doing good work, and is a model of its kind.

THE *Medical Record* says: Dr. Gunn's Medical College Bill was very properly vetoed by the Governor. The strongest argument Dr. Gunn made for his institution was that he believed he could do as well as some other colleges in the State.

TURPENTINE IN THE TREATMENT OF DYSENTERY.—Genkin ("Wratsch;" "Dtsch. Med.-Ztg.") commends the use of oil of turpentine, in doses of ten drops to a teaspoonful of castor oil, and states that he has produced better results with it than by using opium. In only seven out of fifty-nine cases was there any disturbance of the urinary organs.—*N. Y. Medical Journal.*

CAN'T BE TRUSTED.—"I tell you, sir, no woman can be fully trusted," exclaimed a cynical man to a friend. "Why, just look at poor Sniffson. Didn't he love that wife of his? Didn't he think that nothing was too good for her? And how she has requited him!" "How?" asked the other. "Gone and had twins—these hard times."

MURIATE OF COCAINE.—Dr. A. B. Lyons, in the *Weekly Drug News*, has this to say of this import agent: "There is in the market to-day an article labeled "cocaine muriate," which contains either no alkaloid at all or the merest trace of alkaloid—not sufficient to identify it as cocaine, when the quantity operated upon is only a gramme or two. It produces no numbness of the tongue; it leaves an ash, on igniting, amounting to 85 per cent. of the whole, and consisting chiefly of sodium carbonate. It effervesces strongly itself on contact with an acid, showing that it is a carbonate. It contains, however, also notable quantities of chloride and sulphate." This should cause physicians to be exceedingly careful in the purchase of cocaine, and to accept none that does not bear the label of some responsible house. We are satisfied that they can always get it pure from Parke, Davis & Co., of Detroit, a firm that would not furnish an impure article of anything.

A GOOD MOVE.—At a recent session of the Medical Association of Georgia, the ATLANTA MEDICAL AND SURGICAL JOURNAL was adopted as the journal of the Association, and the Transactions of the Association will be published henceforward in that journal.—*Southern Practitioner.*

JEFFERSON MEDICAL COLLEGE.—Prof. J. W. Holland, of Louisville, has been elected to the Chair of Chemistry, *vice* Prof. Mallette, resigned. It is announced that Prof. W. H. Pancoast has tendered his resignation from the Chair of Anatomy, but as yet no action has been taken upon it by the Board of Trustees. The Faculty has discontinued the Post-Graduate Course.

SANITARY Suggestions, or, How to Disinfect our Homes, is the title of a well written pamphlet of 58 pages by B. W. Palmer, A. M., M. D., published by Geo. S. Davis, Detroit; price 25 cts. It contains many valuable suggestions for the use of disinfectants. It is intended for popular perusal, and should have a wide circulation.

THE name of the Atlanta Medical and Surgical Union has been changed to the Atlanta Society of Medicine. The Society has recently refurnished their hall, and have opened a reading room in which all the latest medical journals may be found. The reading room is a new feature of the Society and will add greatly to its usefulness.

DR. ROBERT BATTEY, of Rome, recently made five ovariotomies in one week.

VALUABLE GIFTS.—The following books have been donated to the library of the Atlanta Society of Medicine during the week ending June 20th, 1885:

From P. Blakiston, Son & Co., Philadelphia: Diseases of Children, Day; Hand-book of Skin Diseases, Von Harlengen; Chronic Bronchitis, Greenhow; Dental Caries and its Causes, Leber and Rottenstein; Pennsylvania Hospital Reports; Diseases of the Liver, Harley; Laryngeal Growths, Mackenzie; Diseases of Children, Hillier; Sydenham Society Reports on Medicine and Surgery; John Hunter and His Pupils, Gross. From J. B. Lippincott & Co., Philadelphia: Science and Practice of Medicine, 2 vols., Aitken; DaCosta's Medical Diagnosis; Therapeutics, Materia Medica and Toxicology, Wood; Practical Microscopy, Davis; Pronouncing Medical Dictionary, Thomas; Hand-book of Vaccination, Seaton. At the last meeting of the Society, a unanimous vote of thanks was tendered the above publishers for their liberal and valuable contributions.

MENSTRUATION IN AN INFANT.—Dr. V. Derveer (*Il Morgagni Lancet*) relates the case of an infant girl, who, when barely four months old, commenced to menstruate regularly. When two years and seven months old she weighed forty pounds. She had the facial expression and physical conformation of a girl from ten to twelve years of age. The mammæ were of the size of small oranges, the mons veneris well developed and covered with hair, the labia majora and other parts of the vulva fairly developed. The child was very intelligent, but of an irritable temperament, which was especially marked at the approach of the catamenial periods. These recurred as a rule every four weeks and lasted four or five days, but at one time were interrupted for three months. The child was then exceedingly irritable, and had sleepless nights, but perfect health was restored as soon as the menstrual regularity was re-established.—*Jour. Am. Medical Association.*

DRAMATIS PERSONÆ.—Gynæcologist and Patient, who had married a widower with several children, one of whom was in the waiting-room. *Gynæcologist*, looking through the speculum—"How many children have you?" *Patient*—"We have four in the family, doctor." "Ah! four children. That explains the condition of your cervix, madam. It was badly lacerated at your last confinement, and can only be relieved by trachelorrhaphy." "But, doctor, ain't you mistaken? I—" "Mistaken, madam! Impossible. I tell you, you have laceration of the cervix, dating from your last confinement." "But, doctor—" "Now, madam, I know what is the matter with you, and it's no use for you to volunteer any further information. You must submit to an operation." "But, doctor, I *will* speak. I never had a child. The children we have are my husband's by a former marriage." Tableau.—*Medical Age.*

THE cause of the outbreak of cholera in Spain, says a correspondent of the *British Medical Journal*, is not very difficult to get at. Burjasot lies a little to the northwest of Valencia, on rather high ground, and has a canal taken from the Turia, which flows around the northern part of the town. This is used as the great washing centre, not only for the town, but for the clothes of the Valencians. Surrounding the town in every direction are piled huge heaps of all sorts of animal and vegetable garbage, the scavenging of Valencia. The wells attached to the houses are deep; the water is bad; stables are in close proximity. The people have not the slightest idea of what water contamination means. I find it a thankless task, and often a hopeless one, to produce an impression on my most respectable patients. Now that they find the disease is really on them, all is panic, and no doubt this city will be emptied of all the people that can escape.

THE twenty-eighth annual announcement of the Atlanta Medical College—session 1885–6 has just been issued.

DR. and Mrs. H. V. M. Miller left the city June 30 for Haywood White Sulphur Springs, in Western North Carolina, where they will spend the summer.

DR. J. W. STONE, of Detroit, the popular representative of Parke, Davis & Co., gave us a pleasant call a few days since. He has been canvassing the physicians of Georgia, Alabama and Louisiana in the interest of his house, and says: "I find THE ATLANTA MEDICAL AND SURGICAL JOURNAL wherever I go."

PEOPLE generally, and even those who may be termed steady readers and close observers, have but a faint conception of the magnitude and influence the press of this country has attained. From a careful examination of the 1885 edition of the *American Newspaper Directory*, issued May 1st by Geo. P. Rowell & Co., of New York, it appears that there are 14,147 newspapers and periodicals published in the United States and Canada; of these the United States has 12,973, an average of one paper for every 3,867 persons.

WE call attention to the advertisement of the Piedmont Air-Line, which appears in this issue of THE JOURNAL—last page. This line is one of the best managed and equipped in the South, forming the shortest and most direct route between the South and North, and reaching some of the most delightful summer resorts in the Carolinas and Virginia. It operates and controls the *Western North Carolina Railroad*, which penetrates that grand and beautiful mountain country lying in the western portion of the State, celebrated for its beautiful mountain scenery and healthful climate. This part of Western North Carolina has been appropriately called the "Land of the Sky," and must be seen to be appreciated.

